# Cancer-associated mesenchymal stem cells aggravate tumor progression

**DOI:** 10.3389/fcell.2015.00023

**Published:** 2015-04-01

**Authors:** Chie Kudo-Saito

**Affiliations:** Institute for Advanced Medical Research, Keio University School of MedicineTokyo, Japan

**Keywords:** mesenchymal stem cell, cancer, metastasis, epithelial-to-mesenchymal transition, immunosuppression

## Abstract

Mesenchymal stem cells (MSCs) have both stemness and multi-modulatory activities on other cells, and the immunosuppressive and tumor-promotive mechanisms have been intensively investigated in cancer. The role of MSCs appears to be revealed in tumor aggravation, and targeting MSCs seems to be a promising strategy for treating cancer patients. However, it is still impractical in clinical therapy, since the precise MSCs are poorly understood in the *in vivo* setting. In previous studies, MSCs were obtained from different sources, and were prepared by *ex vivo* expansion for a long term. The inconsistent experimental conditions made the *in vivo* MSCs obscure. To define the MSCs in the host is a priority issue for targeting MSCs in cancer therapy. We recently identified a unique subpopulation of MSCs increasing in mice and human with cancer metastasis. These MSCs are specifically expanded by metastatic tumor cells, and promote tumor progression and dissemination accompanied by immune suppression and dysfunction in the host, more powerfully than normal MSCs growing without interference of cancer. In this review, we summarize current knowledge of the role of MSCs in tumor aggravation, along with our new findings of the bizarre MSCs.

## Introduction

Mesenchymal stem cells (MSCs) have stemness including self-renewability and pluripotency to differentiate into adipocytes, osteocytes, chondrocytes, fibroblasts, pericytes and more, and also have multiple immunoregulatory properties for maintaining immune tolerance (Uccelli et al., 2008; Liu et al., 2009). MSCs suppress immune responses directly by producing immunomodulatory molecules such as IDO, PGE2, TGFβ and nitric oxide, and indirectly via generation and expansion of potent immunosuppressors such as CD4^+^FOXP3^+^ or CD8^+^FOXP3^+^ regulatory T cells (Tregs) and myeloid-derived regulatory cells including dendritic cells (DCregs), monocytes/macrophages (M-MDSCs) and granulocytes (G-MDSCs) (Nauta and Fibbe, 2007; Uccelli et al., 2008; Maggini et al., 2010). Recent studies have demonstrated that MSCs are originally silent, and become immunosuppressive by activation with pro-inflammatory cytokines such as IFNγ, TNFα, and IL-1β, and ligation of TLRs such as TLR2, TLR3, and TLR4 expressed on the MSCs (Burr et al., 2013; English, 2013). This suggests that the functional role of MSCs depends on the components within the microenvironment.

Tumor tissues contain a number of influential factors for recruiting and activating MSCs (Uccelli et al., 2008; Yang et al., 2013). In turn, in the tumor microenvironment, the MSCs modulate biological properties of tumor cells directly by causing epithelial-to-mesenchymal transition (EMT) followed by induction of tumor metastasis and cancer-initiating stem cells (CSCs), and indirectly via angiogenesis followed by promotion of tumor growth (Uccelli et al., 2008; Yang et al., 2013). These totally lead to tumor heterogeneity responsible for resistance to various treatments. Thus, targeting immunoregulatory and tumor-promotive MSCs seems to be a promising strategy for both attenuating the tumor malignancy, and improving the host immunity against cancer in treating patients.

However, targeting MSCs is still unrealistic in clinical settings, because the *in vivo* MSC profiles remain obscure. The surface molecules expressed in MSCs have been widely investigated, and many studies demonstrated the high expression of CD49a, CD73, CD90, CD105, CD146, CD271, and STRO-1, but not CD11b, CD14, CD19, CD34, CD45, CD79a in human MSCs (Nauta and Fibbe, 2007; Kuhn and Tuan, 2010). However, these molecules are not unique markers specific for MSC phenotype and function. In previous studies, MSCs were mostly obtained from different sources followed by *ex vivo* expansion for a long term. This implies that only a part of MSC subpopulations might be selectively expanded, or transformation in MSCs might generate another type different from the original MSCs in the host. Furthermore, the biological properties of MSCs may be much more modulated under cancer than we have ever known in regenerative research without cancer. These inconsistent experimental conditions impede development of targeting MSCs in cancer therapy. To rigorously define the precise MSCs in cancer patients is a priority issue for the practical application of MSC targeting in the treatment.

We have been exploring novel anticancer therapeutics by focusing on the interplay between neoplastic lesions and host immunity for a while, and have recently provided new insights into the metastatic process (Kudo-Saito, 2013; Kudo-Saito et al., 2013a, 2014). Using murine and human tumor cells with typical features of EMT, high motility and invasivity, following transduction of a cDNA coding for snail family zinc finger 1 (Snail), we found that Snail^+^ metastatic tumor cells specifically release a large amount of TSP1 (Kudo-Saito et al., 2009), CCL2 (Kudo-Saito et al., 2013b), and FSTL1 (Kudo-Saito, 2013; Kudo-Saito et al., 2013a), all of which can generate immune suppression and dysfunction mediated by immunoregulatory cells including CD4^+^Foxp3^+^ Tregs, CD11c^+^MHC II^low/−^ DCregs and CD45^−^ALCAM^+^ MSCs, and functionally impaired CD8^low^ T cells. This pathway totally accelerates cancer metastasis in the host. Among the Snail^+^ tumor-producing factors, FSTL1, which is a member of the SPARC family (Sylva et al., 2013), is an outstanding molecule playing a dual role in cancer metastasis particularly to the bones. FSTL1 confers the invasive potential and bone tropism on tumor cells, and also generates and expands CD45^−^ALCAM^+^ MSCs initially in bone marrow and sequentially all over the body in the host (Kudo-Saito, 2013; Kudo-Saito et al., 2013a). We compared the biological properties between Snail/FSTL1-expanded MSCs (designated “sMSCs”) and other MSCs manipulated by none or non-metastatic tumor cells prepared *in vitro* and *in vivo*. The *in vitro* MSCs were CD45^−^ cells sorted from bone marrow, spleen and peripheral blood of naive mice followed by stimulation with none or culture supernatant of Snail^−^ or Snail^+^ tumor cells for 5–7 days. The *in vivo* MSCs were CD45^−^ cells freshly isolated from those tissues of naive mice or the mice implanted with Snail^−^ or Snail^+^ tumor cells (labeled with GFP for elimination). We found that the sMSCs prepared either *in vitro* or *in vivo* specifically produce ANGPT2, CCL2, CCL3, and FSTL1, and most powerfully affect tumor behavior and host immunity by using these molecules (unpublished data except CCL2). ALCAM is expressed only in these MSCs, but not in normal MSCs growing without interference of cancer. These MSCs could be the “activated MSCs” with immunoregulatory properties, since ALCAM is known as an activation marker as well as a MSC marker (Weidle et al., 2010). We validated these results observed in the murine system, using human system with human tumor cells and PBMCs. In addition, we immunohistochemically analyzed tumor tissues of advanced breast cancer patients, and found that accumulation of ALCAM^+^ cells (possibly including the sMSCs) significantly correlates with FSTL1 expression level in tumor portions, but not in the adjacent normal counterparts. This points to the *in vivo* existence of a causal molecular connection between FSTL1 and ALCAM within the tumor microenvironment in patients.

**Figure 1:**
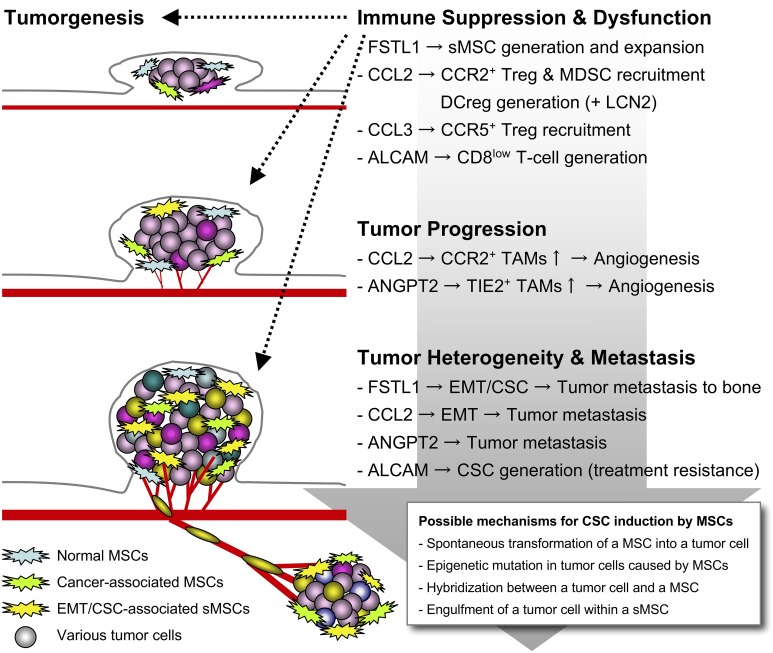
**Tumor aggravation caused by the sMSCs**. When EMT happens in tumor cells, the Snail^+^ metastatic tumor cells generate and expand multifunctional sMSCs that highly release FSTL1, CCL2, CCL3, and ANGPT2 for potentiating tumor aggravation. The sMSCs promote tumor progression and metastasis by building tumor heterogeneity within the microenvironment, and induce immune suppression and dysfunction for further accelerating the tumor survival and escape.

In another study focusing on human endogenous retrovirus antigen H (HERV-H) that is frequently and highly expressed in metastatic tumor cells, we also found that CD45^−^CD271^+^ MSCs are specifically recruited by CCL19 released from HERV-H^+^ tumor cells in the microenvironment (Kudo-Saito et al., 2014). These MSCs are expanded by a 17-mer peptide that is encoded in the immunosuppressive domain of the HERV-H envelope protein. HERV-H was also found to upstreamly regulate Snail and Twist expressions in the metastatic tumor cells. In the immunohistochemical analysis, the significant correlation between HERV-H and CCL19 expressions and accumulation of CD271^+^ cells (possibly including the HERV-H-induced MSCs) was observed in tumor tissues of colon cancer patients. This also suggests the clinical relevance of the experimental findings. Later, we additionally found a high similarity of the phenotype and function between the sMSCs and the HERV-H-induced MSCs. Both ALCAM and CD271 are the functional molecules required for cell proliferation and sphere formation of these MSCs, and the soluble factors released by the sMSCs are also upregulated in the HERV-H-induced MSCs (unpublished data). This suggests that the CD45^−^ALCAM^+^CD271^+^ MSCs (also designated “sMSCs” below) could be the cancer-activated MSCs *in vivo*, particularly generated and expanded in the presence of cancer metastasis in patients. The sMSCs would be a promising target for breaking through the difficulty in treating patients with cancer metastasis and impaired immunity. Here, we summarize the current knowledge of the diversified functional role of the bizarre sMSCs (Figure 1) including normal MSCs in tumor aggravation.

## MSCs promote tumor growth

Because of the high migratory property, MSCs promptly migrate into tumor tissues in response to the inflammatory molecules including chemokines such as CCL5 (Karnoub et al., 2007; Luo et al., 2014) and CXCL16 (Jung et al., 2013). MSCs were considered to contribute to tumor progression by causing angiogenesis essential for tumor survival and growth after differentiation into the cancer-associated cells such as fibroblasts (CAFs), pericytes and endothelial cells (Bergfeld and Declerck, 2010). However, the role of the MSC itself begins to be recognized in tumor progression. One paper has demonstrated that CD44 expressed in MSCs is a functional molecule crucial for the migratory and angiogenic properties of the differentiated CAFs (Spaeth et al., 2013). This suggests that the functional role of fibroblasts depend on the original MSCs. In our study, a number of angiogenic factors were significantly upregulated in the sMSCs, and also in the MSCs manipulated by non-metastatic tumor cells as compared to normal MSCs. This seems to be the cancer-associated features. Particularly, ANGPT2 and CCL2 were specifically released by the sMSCs leading to the much more aggressive tumor growth, although no superior production of other potent angiogenic molecules like VEGFs was seen in the sMSCs compared to other MSCs (unpublished data). When the sMSCs were transfected with siRNAs specific for angpt2 or ccl2 before coinjection with tumor cells in mice, the sMSC-induced tumor growth accompanied by increase of F4/80^+^TIE2^+^ tumor-associated macrophages (TAMs) or CD11b^+^Gr1^+^ MDSCs in the tumor tissues was significantly inhibited. This suggests that the sMSC-derived ANGPT2 and CCL2 play a key role in the sMSC-induced tumor growth. ANGPT2, which binds to TEK/TIE2, is the antagonist of a potent angiogenic molecule ANGPT1 (Fagiani and Christofori, 2013). However, as well as CCL2, ANGPT2 has been demonstrated to promote tumor growth indirectly via increase of TIE2^+^ TAM-like macrophages in the tumor microenvironment (De Palma and Naldini, 2011). FSTL1 was reported to significantly increase in CAFs within stroma of colon cancer tissues (Torres et al., 2013). However, FSTL1 was a small impact on tumor growth in our study.

Some studies have reported no MSC efficacy on tumor growth (Bergfeld and Declerck, 2010). One possible reason for the paradoxical effects may be the experimental conditions such as MSC sources and *in vitro* culture term. Another possible reason may be the distribution site and the amount of the MSCs *in vivo*. In our experiments, the sMSC effect on tumor growth depended on the route of injection of MSCs into mice. Subcutaneous tumor growth was much more aggressively promoted by subcutaneous (s.c.) coinjection with the sMSCs as compared to the case of intravenous (i.v.) injection, by which only a small and limited amount of the sMSCs spontaneously migrated into the tumor tissues (Kudo-Saito et al., 2013a).

## MSCs promote tumor metastasis

Recent studies begin to uncover the roles of MSCs in facilitating tumor metastasis. CCL5 is one of the chemokines produced from both tumor cells and MSCs in the tumor tissues of breast cancer (Karnoub et al., 2007) and prostate cancer (Luo et al., 2014). Tumor-derived CCL5 recruits MSCs to the microenvironment, and the MSCs also release CCL5 for inducing EMT in the tumor cells. Possibly because the major source of MSCs is bone marrow, the relationship between MSCs and bone metastasis has been intensively investigated (Bergfeld and Declerck, 2010). Bone metastasis is frequently seen in patients, particularly with breast cancers and prostate cancers, giving a high risk that deteriorates the quality of life of patients leading to poor prognosis (Weilbaecher et al., 2011). Previous studies demonstrated that tumor cells migrate into bone marrow in response to various chemokines and cytokines such as CXCL12, CCL2, and IL6, which are released by MSCs (Bergfeld and Declerck, 2010). However, little is known about the molecular mechanisms how MSCs direct tumor cells to the bone.

In our study, we did not observed the significant production of CCL5 and CXCL12 in the sMSCs. Instead, ANGPT2, CCL2, and FSTL1 are significantly upregulated in the sMSCs compared to other MSCs (Kudo-Saito et al., 2013a). The crucial roles of ANGPT2 (Minami et al., 2013; Rigamonti and De Palma, 2013) and CCL2 (Lu et al., 2009; Tang and Tsai, 2012) have been already established in the mechanism of cancer metastasis. We also demonstrated the significance of CCL2 in EMT-associated cancer metastasis (Kudo-Saito et al., 2013b). Later, we additionally found that FSTL1 plays a key role in bone metastasis. FSTL1 can confer bone polarity on tumor cells by inducing the bone metastasis-associated molecular expression such as CCR2, CXCR4, and RANKL (Kudo-Saito et al., 2013a). Cancer metastasis is amplified by CCL2 and FSTL1, which are abundantly produced by both the sMSCs and the metastatic tumor cells within the tumor microenvironment. Interestingly, in addition to the soluble factors, cell-cell contact between tumor cells and the sMSCs is required for tumor metastasis, particularly to bone marrow, where is a niche for maintaining CSCs (Kudo-Saito et al., 2013a). This is also involved in the mechanism of the sMSC-created tumor heterogeneity as described below.

## MSCs create tumor heterogeneity and complexity

It is increasingly being recognized that every tumor cell has a different profile in gene expression and cellular function even if the source (tumor tissue and patient) is just same, and the clonal evolution in a tumor cell builds tumor heterogeneity and complexity governing resistance to the treatments leading to poor outcome in patients (Junttila and De Sauvage, 2013). Although the extracellular matrix is one of the most influential factors for altering tumor properties (Faurobert et al., 2015), MSCs have been also demonstrated to play a key role in generating CSCs with a high metastaticity, dormancy and chemoresistance. In our study, we also observed that tumor cells transform into CSC-like tumor cells following *in vitro* coculture or *in vivo* coinjection with the sMSCs (Kudo-Saito et al., 2013a). This is partly mediated by cell-cell interaction through the ALCAM-ALCAM homophilic binding after ALCAM clustering on the cell surface, which is crucial for proliferation and colonization of CSCs (Weidle et al., 2010) and the sMSCs (Kudo-Saito et al., 2013a). Interestingly, Roodhart et al. reported platinum-based chemotherapy activates MSCs to produce polyunsaturated fatty acids, which confer chemoresistance on tumor cells (Roodhart et al., 2011). This gives a warning in practical settings of treating cancer patients with chemotherapeutics.

In addition to such epigenetic mutations in tumor cells, spontaneous hybridization between tumor cells and MSCs has been recently demonstrated as a non-mutational mechanism in breast cancer (Rappa et al., 2012) and lung cancer (Xu et al., 2014). The tumor/MSC-fused cells have CSC-like properties such as high tumorigenicity and metastaticity, maintaining both profiles of tumor cells and MSCs. In our study, when tumor cells were cocultured with the sMSCs in phagocytosis assay, a few tumor cells were engulfed by the sMSCs, but not fused together, and the tumor cells taken were kept inside of the sMSCs alive without cell death or digestion for a long term (unpublished data). This may be another mechanism that tumor cells lurk with the help of MSCs in the host. To analyze the phenotype and behavior of these tumor-holding MSCs may contribute to further understanding of the mechanism involved in tumor survival and escape. Interestingly, it has been also demonstrated that MSCs transform into tumor-like cells with high proliferative ability following spontaneous mutations (Miura et al., 2006; Tolar et al., 2007; Rosland et al., 2009; Mohseny and Hogendoorn, 2011). This seems to be the fundamental mechanism underlying the emergence of the cancer stem cells originated from non-cancerous cells in the host.

## MSCs protect tumor cells from immune attack

MSCs cause immune suppression and dysfunction, which totally support all steps of tumor progression (Nauta and Fibbe, 2007; Uccelli et al., 2008). Such immunoregulatory MSCs have been interest in the therapy to properly induce and activate anti-tumor immune responses in cancer patients, since impaired immunity has been a critical issue in the treatment (Uccelli et al., 2008; Yang et al., 2013). Recent studies have demonstrated that MSCs become immunosuppressive following activation through inflammatory signals (Burr et al., 2013; English, 2013). A tumor tissue, where is a milieu filled with abundant pro-inflammatory molecules produced by tumor cells and a variety of the infiltrating cells, could be the best educational place for MSCs to acquire the distinctive competence to support tumor cells. However, how to distinguish between the cancer-activated MSCs and normal healthy MSCs remains to be elucidated.

In our study, we identified CCL2, CCL3, and FSTL1 as the prominent immunomodulatory molecules upregulated only in the sMSCs. The sMSCs generate functionally impaired CD8^low^ T cells through cell-cell contact with ALCAM expressed on the sMSCs (Kudo-Saito et al., 2013a), in addition to regular Tregs as reported elsewhere (Nauta and Fibbe, 2007; Uccelli et al., 2008; Maggini et al., 2010). In the previous studies showing Treg or CD8^low^ T-cell induction by MSCs, CD8 reduction in the Foxp3^+^ T cells (Prevosto et al., 2007), or Foxp3 expression in the CD8^low^ T cells (Giuliani et al., 2011) were not analyzed. The sMSC-derived FSTL1 might amplify such abnormal immunity by promoting the sMSC expansion in an autocrine manner as well as a paracrine manner by the metastatic tumor-derived FSTL1. CCL2 is also responsible for immunosuppression. In our study, CCL2 orchestrates CCR2-expressing immunomodulatory cells including Tregs, MDSCs and TAMs in the tumor microenvironment, and generates DCregs partly in collaboration with LCN2 that is released from the CCL2-stimulated tumor cells (Kudo-Saito et al., 2013b). Tregs within tumor microenvironment have been demonstrated to highly express a CCL3 receptor, CCR5 (Tan et al., 2009; Schlecker et al., 2012). The sMSC-derived CCL3 may also help to promptly create a beneficial environment for immunoevasion of tumor cells by recruiting the cancer-associated CCR5^+^ Tregs, although not yet investigated in our study.

## Concluding remarks

The significant advances in profiling MSCs provided abundant knowledge about MSCs, and revealed the central role of MSCs in tumor development and progression. The emerging evidences show that MSCs stand out as a promising therapeutic target for treating cancer and the consequent impaired immunity in patients. Nevertheless, we have understood little about the MSCs interacting with tumor cells in the *in vivo* setting. Our sMSC study is gradually unmasking the characteristics of the cancer-activated MSCs that can sharply build heterogeneic and complexed microenvironment beneficial for tumor survival and escape in the host. However, further studies are still needed to achieve translation of the findings to the clinical therapy.

### Conflict of interest statement

The author declares that the research was conducted in the absence of any commercial or financial relationships that could be construed as a potential conflict of interest.
